# The novel Akt inhibitor Palomid 529 (P529) enhances the effect of radiotherapy in prostate cancer

**DOI:** 10.1038/sj.bjc.6604938

**Published:** 2009-02-24

**Authors:** R Diaz, P A Nguewa, J A Diaz-Gonzalez, E Hamel, O Gonzalez-Moreno, R Catena, D Serrano, M Redrado, D Sherris, A Calvo

**Affiliations:** 1 Division of Oncology, Center for Applied Medical Research (CIMA). University of Navarra, Pamplona, Spain; 2 Department of Oncology, Clinica Universitaria, University of Navarra, Pamplona, Spain; 3 Division of Cancer Treatment and Diagnosis, Toxicology and Pharmacology Branch, National Cancer Institute at Frederick, National Institutes of Health, Frederick, MD 21702, USA; 4 Paloma Pharmaceuticals, Jamaica Plain, MA, USA

**Keywords:** prostate cancer, radioresistance, Akt/PKB activation, VEGF, Id-1, Palomid 529

## Abstract

Radiotherapy (RT) is a common treatment for localised prostate cancer, but can cause important side effects. The therapeutic efficacy of RT can be enhanced by pharmacological compounds that target specific pathways involved in cell survival. This would elicit a similar therapeutic response using lower doses of RT and, in turn, reducing side effects. This study describes the antitumour activity of the novel Akt inhibitor 8-(1-Hydroxy-ethyl)-2-methoxy-3-(4-methoxy-benzyloxy)-benzo[*c*]chromen-6-one (Palomid 529 or P529) as well as its ability to decrease radiation-activated phospho-Akt (p-Akt) signalling in a prostate cancer model. P529 showed a potent antiproliferative activity in the NCI-60 cell lines panel, with growth inhibitory 50 (GI50) <35 *μ*M. In addition, P529 significantly enhanced the antiproliferative effect of radiation in prostate cancer cells (PC-3). Analysis of signalling pathways targeted by P529 exhibited a decrease in p-Akt, VEGF, MMP-2, MMP-9, and Id-1 levels after radiation treatment. Moreover, the Bcl-2/Bax ratio was also reduced. Treatment of PC-3 tumour-bearing mice with 20 mg kg^−1^ P529 or 6 Gy radiation dose decreased tumour size by 42.9 and 53%, respectively. Combination of both treatments resulted in 77.4% tumour shrinkage. Decreased tumour growth was due to reduced proliferation and increased apoptosis (as assessed by PCNA and caspase-3 immunostaining). Our results show the antitumour efficacy of P529 alone, and as a radiosensitiser, and suggest that this compound could be used in the future to treat human prostate cancer.

Chemotherapy and radiotherapy (RT) are regular treatments to decrease tumour burden and ameliorate tumour-related symptoms. However, current regimes are not curative in most cases, and, in general terms, cancer mortality rates have not decreased significantly in recent years (Jemal *et al*, 2008). This situation has prompted many researchers and companies to develop novel compounds with a higher and more selective antitumour activity. Our study using Palomid P529 (P529) (Paloma Pharmaceuticals, Jamaica Plain, MA, USA) has been focused on prostate cancer, the most common malignancy in male subjects, affecting more than 10% of men over 65 years (Jemal *et al*, 2008). The choice of a therapeutic modality for localised prostate cancer is often dependent on the patient's age, health, lifestyle, and perceived chances for cure and potential complications. Radiotherapy is applied in localised prostate tumours with a curative intention. Dose increase has been associated to higher control rates. Nevertheless, high dose of radiation may have important side effects, such as impotence, urinary dysfunctions, and rectal symptoms (Baxter *et al*, 2005). New therapeutic strategies are needed as well to improve the efficacy of RT in radioresistant patients. The use of drugs with low toxicity that may work as radiosensitisers could solve these problems by reducing radiation dosage while achieving similar therapeutic efficacy, thus improving the therapeutic ratio. Some emerging candidate radiosensitisers for prostate cancer are 2-methoxyestradiol (Casarez *et al*, 2007) and genistein (Raffoul *et al*, 2006), two low toxic compounds with both antiangiogenic and antitumour properties that act synergistically to enhance the effect of RT in animal models of prostate cancer.

In this study, we have used the novel compound 8-(1-Hydroxy-ethyl)-2-methoxy-3-(4-methoxy-benzyloxy)-benzo[*c*]chromen-6-one (Palomid 529, or P529) that has been recently shown to target Akt/mTOR pathways (Xue *et al*, 2008). This drug is a derivative of 3-hydroxy dibenzo[*b*,*d*]pyran-6-one, which was previously described to share structural similarities with the potent antiangiogenic and antitumour compound 2-methoxyestradiol, and to exhibit selective antiproliferative activity for endothelial cells (Schmidt *et al*, 2003). Recently, benzochromenones were reported to differentially suppress the growth of certain tumour cell lines (Dai *et al*, 2007). In addition, P529 showed to reduce tumour growth, angiogenesis, and vascular permeability in an *in vivo* model of glioblastoma (Xue *et al*, 2008).

Our hypothesis was that as P529 inhibits the Akt pathway (Xue *et al*, 2008), which is critically involved in radioresistance (McKenna and Muschel, 2003; Cheng *et al*, 2006) and lacks *in vivo* toxicity (Xue *et al*, 2008), the use of this drug would be an excellent novel candidate to enhance the effect of RT in prostate cancer. Here, we show that, indeed, P529 potentiates the effect of RT in the aggressive prostate cancer cell line, PC-3, mainly through the blockade of p-Akt activation, but also through the alteration of other cancer-related pathways involving MMP-2, MMP-9, Id1, and vascular endothelial growth factor (VEGF). Moreover, we show that P529 enhances the antitumour effect of RT *in vivo* by reducing the proliferation rates and promoting apoptosis.

## Materials and methods

### NCI cell growth inhibitory screening

Palomid 529 (P529) was provided from Paloma Pharmaceuticals Inc. The ability of P529 in inhibiting cell growth proliferation was tested in the NCI-60 tumour cells panel. The antitumour effect was tested by the NCI core facility, following NCI protocols publicly available at the website: http://dtp.nci.nih.gov/docs/compare/compare_methodology.html#specon. Briefly, cells were grown in RPMI 1640 medium containing 5% foetal bovine serum and 2 mM L-glutamine, and then inoculated into 96-well microtitre plates in 100 *μ*l at plating densities ranging from 5000 to 40 000 cells per well, depending on the doubling time of individual cell lines. After 24 h, two plates of each cell line were fixed *in situ* with TCA, to represent a measurement of the cell population for each cell line at the time of drug addition (time zero (Tz)). P529 was applied at different concentrations (solubilised in DMSO). Following drug addition, the plates were incubated for an additional 48 h. For adherent cells, the assay was terminated by the addition of cold TCA. The supernatant was discarded, and the plates were washed five times with tap water and air-dried. Sulforhodamine B (SRB) solution (100 *μ*l) at 0.4 % (w/v) in 1% acetic acid was added to each well, and plates were incubated for 10 min at room temperature. After staining, unbound dye was removed by washing with 1% acetic acid. Bound stain was subsequently solubilised with 10 mM trizma base, and the absorbance was read on an automated plate reader at a wavelength of 515 nm. For suspension cells, the methodology was the same except that the assay was terminated by fixing settled cells at the bottom of the wells by gently adding 50 *μ*l of 80% TCA. Using the seven absorbance measurements (Tz, control growth, (C), and test growth in the presence of drug at the five concentration levels (Ti)), the percentage growth was calculated at each of the drug concentrations levels. Percentage growth inhibition was calculated as follows: 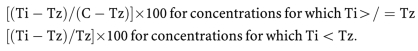 Data are shown as growth inhibition of 50% (GI50), which was calculated from ((Ti−Tz)/(C−Tz)) × 100=50.

### Cell growth inhibition of human umbilical vascular endothelial cell (HUVEC) and PC-3 cells

As dibenzo[*b*,*d*]pyran-6-one derivatives have been shown to inhibit very efficiently endothelial cell growth and the NCI 60 tumour cell panel did not include any endothelial cell type, we tested the antiproliferating effect of P529 on HUVECs by MTT assays. In addition, MTT was also used to confirm the antigrowth effect of P529 in PC-3 cells.

Human umbilical vascular endothelial cells and the required media complements were purchased from Cambrex Bio Science Walkersville Inc (Walkersville, MD, USA). Growth and maintenance of the cultures were carried out as described by the manufacturer. PC-3 cells were cultured in RPMI 1640 medium, supplemented with 10% foetal clone III, and 1% penicilin–streptomycin (all from Invitrogen, Carlsbad, CA, USA). Cultures were incubated at 37°C in a humidified 5% CO_2_ incubator. Proliferation assays for HUVECs were carried out by seeding the cells in 96-well plates at a density of 1000 cells per well. Following a 24-h plating period, cells were cultured for another 24 h in 0.5% serum before being treated with P529 (at 500 nM) in the presence of 100 ng ml^−1^ VEGF. Upon P529 treatment (for 24 and 96 h), proliferation was determined with the MTT Cell Proliferation Kit I (Roche, Mannheim, Germany), according to the manufacturer's recommendations. For PC-3 cells, the assay was performed as follows: 1000 cells were plated in 96-well plates and treated with P529 for 48 h. Cell proliferation rates were determined with the MTT assay as well. Readings were performed at 540/690 nm in the SunRise ELISA plate reader (Tecan Austria GmbH, Salzburg, Austria).

### Effect of P529 on colchicine binding to tubulin

The method was performed according to earlier publications (Ma *et al*, 2007). Each 0.5 ml reaction contained 0.1 M MES (pH 7.0 with NaOH in 1 M stock solution), 0.5 mM MgCl_2_, 1 *μ*M of tubulin [^3^H]colchicine (5 *μ*M), and the indicated concentration of different drugs (1 and 5 *μ*M combretastatin A-4-phosphate (CA4P); 5 and 50 *μ*M 2-methoxyestradiol (2ME_2_); 5 and 50 *μ*M P529). As negative controls, two samples were used: one without tubulin (a blank) and another with tubulin but without inhibitor. Tubulin was added and incubated at 37°C for 120 min in darkness. Reaction mixtures were then added to 1 ml microspin Sephadex G-50 (superfine) columns and processed by centrifugal gel filtration at room temperature. The filtrate was collected, and radioactivity was determined by liquid scintillation counting, which allowed the calculation of a molar ratio of drug bound to tubulin for each experiment. The radioactivity was measured – in counts per minute (CPM) – for each sample. Data were shown as percentage of inhibition (% inhibition), which was calculated from CPM values as follows: 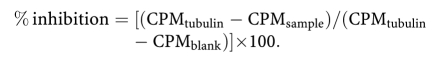


### Tubulin polymerisation assay

The assay was conducted as described previously (Hamel, 2003). Reaction mixtures contained 0.8 M monosodium glutamate, 10 *μ*M tubulin, and different concentrations of P529. Tubulin was preincubated for 15 min at 30°C, and samples were chilled on ice. Guanosine-5′-triphosphate (GTP) (0.4 mM) was added, followed by a temperature jump to 37°C to initiate tubulin polymerisation, which was measured over time at 350 nm in a temperature-controlled recording spectrophotometer for 20 min. Tubulin polymerisation was followed turbidimetrically at 350 nm in Gilford model 250 spectrophotometer (Gilford Instruments Laboratories, Oberlin, OH, USA) equipped with electronic temperature controllers. 2ME_2_ was used as positive control.

### Radiation exposure

PC-3 cells (5 × 10^4^ cells per well) were irradiated at room temperature using 15 MeV electrons (Primus Linear Accelerator, Siemens, Erlangen, Germany) at doses of 2, 4 or 8 Gy. Nonirradiated controls were handled identically to the irradiated cells with the exception of the radiation exposure. After irradiation, cultures were kept at 37°C and 5% CO_2_ in an incubator.

### Proliferative and clonogenic assay after radiation

To investigate the effect of P529 on cancer cells in response to radiation, proliferation, and clonogenic assays were performed. A total of 50 000 PC-3 cells per well were plated (in triplicate) into 6-well plates. After 24 h, cells were treated with 2 *μ*M P529. One day later, cells were irradiated according to the described protocol. After 24 h of irradiation, cells were detached from the Petri dishes with Trypsin-EDTA (Cambrex Bio Science, Verviers, Belgium), resuspended in PBS, and counted in a Neubauer chamber with diluted Trypan Blue (Sigma-Aldrich Inc., St Louis, MO, USA) 1 : 1. Colony formation assays were performed right after, by plating 500 cells per well into 100 mm culture dishes, in triplicates. After 12-day incubation at 37°C and 5% CO_2_, cells were fixed with formaldehyde and stained with 2% crystal violet. The number of colonies was then counted and the surviving fraction was normalised to the surviving fraction of the corresponding control.

### Total protein extraction and western blot

Whole cell pellets were collected 24-h post-irradiation along with the corresponding non-irradiated control groups. Cells were detached from the flasks using Trypsin-EDTA (Cambrex Bio Science), collected by centrifugation and lysed with a lysis buffer (RIPA: 10 mM Tris pH 7,4; 150 mM NaCl; 1% Triton X-100; 1% deoxycholate; 0.1% SDS; 5 mM EDTA). Extracts were aliquoted and stored at −80°C for subsequent western blot analyses. Protein concentrations were determined with the BCA Protein Assay Kit (Perbio, Rockford, IL, USA). In all, 20 *μ*g protein extracts were run through NuPAGE 4–10% Bis-Tris gels (Invitrogen). Electrophoresis was monitored using dual color Precision Plus Protein Standards (BioRad, Hercules, CA, USA). Proteins were transferred to polyvinylidene difluoride membranes (Bio-Rad, Richmond, CA, USA). Membranes were blocked with 5% non-fat dry milk in TBS-Tween (1 × TBS: 0.05 M Tris-HCl, 0.5 M NaCl, pH=7.36; 0.1% Tween-20), and incubated at the recommended dilution with the following antibodies: rabbit anti-Bcl-2 (Santa Cruz Biotechnology, Santa Cruz, CA, USA), rabbit anti-Bax (Santa Cruz Biotechnology), mouse anti-β-actin (Sigma), rabbit anti-Akt (Cell Signaling Technology, Danvers, MA, USA), rabbit anti-phospho-Akt (Cell Signaling Technology), rabbit anti-MMP-2 (Labvision, Fremont, CA, USA), rabbit anti-MMP-9 (Sigma), rabbit anti-VEGF (Santa Cruz Biotechnology), rabbit anti-Id-1 (BioCheck, Foster City, CA, USA). Membranes were then incubated with one of the following secondary antibodies (depending on the species of the primary antibody): goat antimouse IgG (Santa Cruz Biotechnology) or goat antirabbit IgG (Santa Cruz Biotechnology). Immunoblots were developed with the chemiluminescence detection system Lumi-Light PLUS (Roche), exposed to Hyperfilm ECL (Amersham) and developed with an AGFA automated X-ray film processor.

### Animals and therapeutic regimes

Male athymic nude mice were purchased from Harlam (UK). 1 × 10^6^ PC-3 cells were injected s.c. mixed with Matrigel (Becton Dickinson Labware, Bedford, MA, USA) in a final 0.2-ml injection volume. Mice were randomly divided into four groups (six mice per group): (a) controls; (b) treated s.c. with 20 mg kg^−1^ P529 every 3 days; (c) Treated with RT (a single dose of 6 Gy, 1 week after cell injection); (d) combination of P529 and radiation, following similar regimes as described above. The radiation was localised to the tumour. Tumour width (W) and length (L) were measured once a week with a caliper. Tumour volume was calculated as follows: volume (mm^3^)=0.5 × W^2^ × L. Irradiation of mice was carried out 1 week after implantation of the cells, using a single dose of 6 Gy, with a Primus Linear Accelerator (Siemens). Mice were killed 4 weeks after cell injection by inhalation of CO_2,_ and tumour tissues were dissected and then processed for histology. All mice were treated in accordance with the guidelines for the Animal Care Ethics Commission of our institution (University of Navarra) under an approved animal protocol.

### Histology, immunohistochemistry, and quantification of proliferation and apoptosis in tumours

Tissues were fixed in 10% formalin, embedded in paraffin, and sectioned (5-*μ*m thickness). Slides were stained with H&E and Masson's trichrome. For proliferation and apoptosis analyses, immunohistochemistry for PCNA and active caspase-3 was conducted as follows: slides were deparaffinised and incubated for 10 min with 3% H_2_O_2_ in water to quench the endogenous peroxidase activity. An antigen retrieval method was used for the detection of PCNA. Tissues were incubated with 5% normal rabbit serum in TBS (0.05 M Tris-HCl, 0.5 M NaCl, pH=7.36) for 30 min at room temperature. Dilution of the primary antibodies was as follows: 1:100 for mouse monoclonal anti-PCNA (M0879, DakoCytomation Denmark A/S, Glostrup, Denmark) and 1 : 200 for rabbit polyclonal anti-active caspase-3 (Cell Signaling). The indirect avidin–biotin–peroxidase method was applied, using the appropriate secondary antibodies, for 30 min at room temperature. The EnVision (K4007, Dako) signal enhancement system was used to develop the bound antibodies. Sections were counterstained with Harris haematoxylin, dehydrated and mounted. For quantifications, 30 random images ( × 400) per experimental group were captured with a microscope (Leica, Wetzlar, Germany) equipped with the Analysis software. The number PCNA- and caspase-3-positive cells present in the reference area was counted and divided by the total number of cells included in the reference area.

### Statistical analysis

An ANOVA test was used when appropriate to determine significant differences between treatment groups. Statistical significance was considered at the 0.05 and 0.01 levels.

## Results

### P529 inhibits cell growth of a broad spectrum of cancer cell lines

The cell growth inhibitory potential of P529 was first tested in the NCI 60 Cell Screen (Developmental Therapeutics Programe NCI/NIH). P529 inhibited cell growth of virtually all the cell lines tested, with a GI50 lower than 35 *μ*M. The GI50 values were variable: 2–34 *μ*M for leukaemia and non-small cell lung cancer; 6–24 *μ*M for melanoma, colon and ovarian cancers; 2–19 *μ*M for central nervous system (CNS), renal, breast, and prostate cancer, including the PC-3 cell line (Table 1). We conclude that P529 is a novel drug with a wide potential inhibitory growth effect of a variety of tumour types, including prostate cancer.

**Figure 1:**
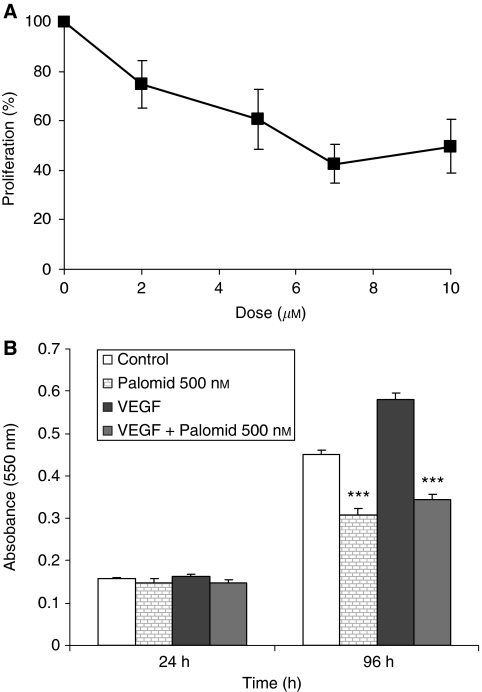
Cytotoxicity of P529 in PC-3 and HUVECs. (**A**) PC-3 cells treated with different doses of P529. The GI50 was determined to be 5–7 *μ*M. (**B**) In HUVECs, 500 nM of P529 in the presence of 100 ng/ml VEGF significantly (*P*<0.001) reduces cell proliferation, 96 h after administration of the drug.

As we were particularly interested in prostate cancer, the growth inhibition of P529 was further tested in the androgen-independent prostate cell line, PC-3, by MTT assays (Figure 1A). Cells were treated with different doses of P529 (0, 2, 5, 7, and 10 *μ*M). P529 caused a concentration dependent growth inhibition on PC-3 cells. Doses of 2 and 7 *μ*M resulted in 30 and 60% growth inhibition, respectively. After 48 h treatment and on the basis of the MTT assay, the GI50 value was estimated to be 5–7 *μ*M (Figure 1A).

Dibenzo[*b*,*d*]pyran-6-one derivatives have been shown to be potent inhibitors of endothelial cell growth, thus suggesting antiangiogenic properties (Schmidt *et al*, 2003). We, therefore, analysed the effect of P529 on HUVECs proliferation. P529 significantly inhibited (*P*<0.001) control and VEGF-stimulated endothelial cell proliferation, 96 h after exposure to the drug (Figure 1B).

### P529 neither affects tubulin polymerisation nor binds to the colchicine site

One of the main mechanisms of the antitumoral drug 2ME_2_ is the inhibition of tubulin polymerisation by interacting with the colchicine site (D’Amato *et al*, 1994). Because of the structural similarities between dibenzo[*b*,*d*]pyran-6-one derivatives and 2ME_2_ (Schmidt *et al*, 2003), we examined whether P529 would bind to tubulin and whether this compound would affect tubulin polymerisation. The displacement of 5 *μ*M [^3^H]colchicine on 1 *μ*M tubulin by P529 or positive control agents was expressed as rate of inhibition. Inhibition rates of P529 at concentrations of 5 or 50 *μ*M were 8 and 7%, respectively, and concentration-independent (Table 2), thus showing a lack of binding to tubulin at the colchicine's site. Both positive controls, CA4P and 2ME_2,_ showed a pronounced concentration-dependent inhibition rates (Table 2). The tubulin-binding assay showed that P529 did not inhibit tubulin polymerisation (results not shown). These data show that the mechanism of action of P529 differs from that of binding to and inhibiting tubulin polymerisation.

### P529 enhances the antiproliferative effect of radiation in PC-3 cells

**Figure 2:**
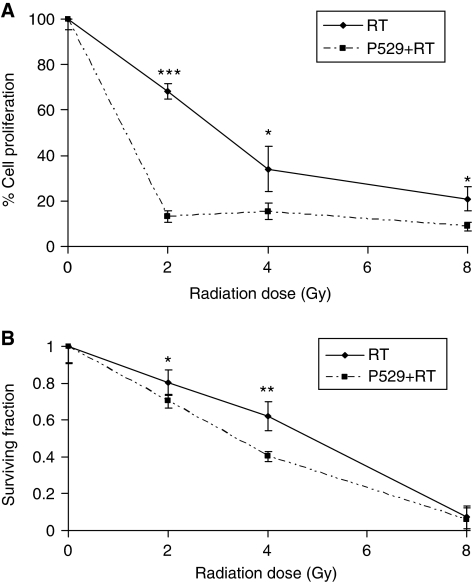
Proliferation and clonogenic assays of RT- and RT+P529-treated cells. (**A**) Administration of 2 Gy to PC-3 cells causes a 30% cell growth inhibition, whereas addition of P529 to 2 Gy radiation results in 85% cell growth inhibition (*P*<0.001). P529 also enhances the antiproliferative effect of 4 Gy radiation (*P*<0.05). (**B**) In clonogenic assays, P529 enhances the effect of RT with significant differences between RT and RT+P529, when 2 and 4 Gy are administered to the cells (*P*<0.05, and *P*<0.01, respectively). ^*^P<0.05; ^**^P<0.01; ^***^P<0.001.

P529 has been previously shown to inhibit Akt signalling (Xue *et al*, 2008). Because inactivation of the Akt-mediated pathway is a demonstrated way to enhance the effect of RT (McKenna and Muschel, 2003; Cheng *et al*, 2006), we investigated whether P529 would increase radiation-induced PC-3 cell growth inhibition. For these experiments, we selected a dose of P529 that had a significant antiproliferative effect on PC-3 cells. As mentioned earlier, treatment of PC-3 cells with 2 *μ*M P529 alone resulted in approximately 30% cell growth inhibition compared with untreated cells, and thus, this concentration was selected for combination purposes. To test the effect of P529 plus radiation, 50 000 PC-3 cells were plated and pretreated with 2 *μ*M P529 for 24 h. Then, cells were irradiated with 0, 2, 4, and 8 Gy. Proliferation of PC-3 cells was analysed 72 h post-plating. In addition, 500 treated cells were plated for a 10-day clonogenic assay. Administration of 2 *μ*M P529 in combination with 2 Gy radiation dramatically decreased the tumour cell proliferation (*P*<0.001), compared with 2 Gy radiation alone (15 *vs* 70% cell survival, respectively, compared with controls). Use of 2 *μ*M P529 plus 4 and 8 Gy radiation also improved significantly the effect of each of the radiation doses alone (*P*<0.05), although the effect was less pronounced (Figure 2A).

In clonogenic assays, reduction of clonogenic capacity was observed with the combination of P529 and RT *vs* RT alone with 4 Gy (40 *vs* 60% survival fraction, respectively; *P*<0.01; Figure 2B). Although the effect was mild, a similar result was found when using 2 Gy dose (*P*<0.05). No differences were observed for high doses of radiation (8 Gy; Figure 2B). These data show that pre-treatment with low doses of P529 sensitises prostate cancer cells to radiation-induced cell growth inhibition.

### P529 inhibits the radiation-induced p-Akt activation and decreases Bcl-2/Bax ratio in PC-3

The PI3K/Akt signalling pathway in cancer cells promotes proliferation, invasiveness, and angiogenesis, thus constituting a critical cell survival mechanism. Activation of this pathway plays an essential role in the resistance to conventional radiation treatment (Cheng *et al*, 2006). The mitochondrial Bcl-2/Bax apoptotic pathway also determines cell fate. To confirm our hypothesis that pre-treatment of PC-3 cells with P529 might reduce radiation resistance of cancer cells, we analysed p-Akt, total Akt, Bcl-2, and Bax protein levels after individual or combined treatments.

**Figure 3:**
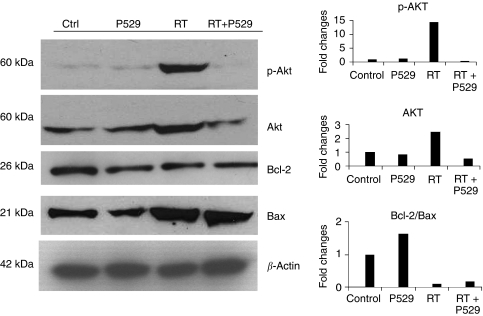
Effect of P529, RT, and P529+RT and the combination on p-Akt, total Akt, Bcl-2, and Bax. Ionising radiation causes a >10-fold increase in p-Akt levels. However, when P529 is added to the treatment, p-Akt levels are lower than those observed in untreated cells. Total Akt levels are also increased in RT-treated cells, but levels return to normal when the drug is added. Bcl-2 decreases as a consequence of P529, RT, and combination treatment, whereas Bax increases in RT- and RT+P529-treated cells. The densitometric analysis shows that the Bcl-2/Bax ratio is reduced in RT and RT+P529 cells. The *Y*-axis represents fold changes with respect to control.

Treatment of cells with 2 Gy radiation increased the levels of total Akt (Figure 3). The active form of Akt (p-Akt) was dramatically increased by 2 Gy radiation (RT; Figure 3). Strikingly, the combination of P529+2 Gy radiation reduced the amount of total and phosphorylated Akt below the control levels (Figure 3). In addition, PC-3 cells treated with P529, radiation, and the combination of both showed reduced levels of the antiapoptotic protein Bcl-2 in comparison with controls (untreated cells). Levels of the proapoptotic protein Bax were higher in RT- and RT+P529-treated cells than in P529 or control cells (Figure 3). Densitometric analysis showed that the Bcl-2/Bax ratio was reduced in cells receiving radiation and RT+P529, compared with control or P529 treatment alone (Figure 3).

### P529 inhibits radiation-induced overexpression of Id-1 and VEGF

**Figure 4:**
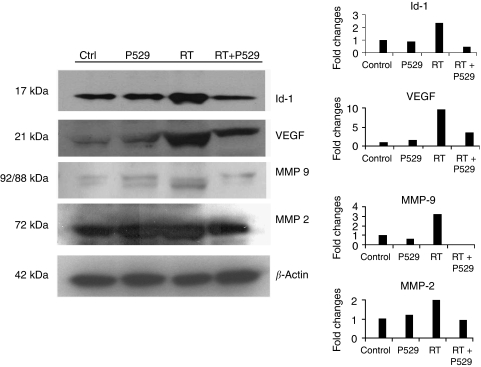
Effect of P529, ionising radiation, and the combination of both treatments on Id-1, VEGF, MMP-9, and MMP-2 protein levels. Amount of Id-1 is increased by RT, but addition of P529 reduces Id-1 below control levels. The combination RT+P529 also decreases RT-induced VEGF levels. Active MMP-9 is reduced in RT-treated cells by the addition of P529. Levels of active MMP-2 are also decreased by the addition of P529 to RT-treated cells. In the densitometric analysis, the *Y*-axis represents fold changes with respect to control.

The overexpression of the inhibitor of differentiation-1 (Id-1) has been related to radioresistance (Zhang and Rosdahl, 2003). Activation of Id-1-mediated pathways leads to the overexpression of the progangiogenic growth factor, VEGF (Ling *et al*, 2006). As P529 has been previously shown to inhibit angiogenesis *in vivo* (Xue *et al*, 2008), we analysed whether treatment of PC-3 cells with this drug would block VEGF/Id-1-angiogenic pathways. Administration of 2 *μ*M P529 alone did not change Id-1 levels, whereas treatment with ionising radiation elevated Id-1 levels (Figure 4). The RT+P529 combination treatment resulted in a reduction of Id-1 below control levels (Figure 4). The overexpression of VEGF by tumour cells is a good example of the induction of prosurvival pathways induced by RT (Berse *et al*, 1992; Gorski *et al*, 1999; Gupta *et al*, 2002). Another consequence of VEGF overexpression is the activation of proangiogenic signalling cascades (including Akt) in the endothelial cells of the tumour vascular bed (Leung *et al*, 1989; Folkman, 1995). Exposure of PC-3 cells to 2 *μ*M P529 slightly increased VEGF levels, whereas RT resulted in a strong VEGF induction. However, addition of P529 to RT reduced VEGF levels (Figure 4). These data show that levels of two critical prosurvival factors (Id-1 and VEGF), whose expression is induced by RT, are decreased by the addition of P529.

### Downregulation of radiation-induced MMP-2 and MMP-9 by P529

Matrix metalloproteases (MMPs) are involved in invasiveness and metastasis (Cheng *et al*, 2006). MMP-2 and MMP-9 levels increase in irradiated cells (Wang *et al*, 2000; Cheng *et al*, 2006). We measured inactive pro-MMP-9 (MW=92 KDa) and active MMP-9 (MW=88 KDa) levels in PC-3 cells after P529, radiation, or P529 plus radiation treatments. In control and P529-treated cells, low levels of inactive and active MMP-9 were found, whereas an increase in the cleaved (active) form was observed after radiation treatment. The combination of P529 with RT decreased both active and inactive MMP-9 levels below control levels (Figure 4). Radiation treatment increased modestly active (72 kDa) MMP-2 levels. However, when radiation was combined with 2 *μ*M P529, levels were found to be similar to those of controls or P529-treated cells alone (Figure 4). The inactive form of MMP-2 did not change with any of the treatments (results not shown). The above findings indicate that P529 blocks radiation-mediated induction of MMP-2 and MMP-9.

### P529 treatment reduces tumour growth and enhances the effect of RT *in vivo*

**Figure 5:**
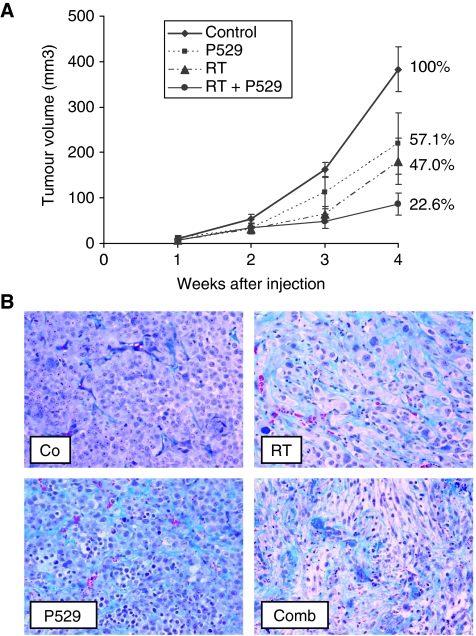
Effect of different treatment regimes in tumour-bearing mice. (**A**) Tumour volume is greatly reduced in P529+RT- treated mice (77.4% reduction). (**B**) Histology of tumours treated with radiotherapy (RT), P529, and combination (Comb), compared with untreated mice (Co). An intense fibrosis and tumour cell damage is observed in mice treated with P529 plus RT.

Treatment of PC-3 tumour-bearing mice with P529 reduced tumour growth to 57.1% compared with controls. Radiotherapy (single dose of 6 Gy) also resulted in a decreased tumour growth (47.0% compared with controls; Figure 5A). Combination of both therapies gave rise to tumours 22.6% in size with respect to untreated mice (77.4% reduction of tumour growth; Figure 5A). No weight loss was observed in any of the experimental groups. Tumours from control mice were characterised by dense cellular content and little stroma. Tumours from irradiated mice showed large areas of cell damage characterised by cell swelling and increased fibrosis. Tumour from P529-treated mice showed cells with picnotic nuclei, and in some instances cytoplasmic swelling. Tumours of mice treated with P529+RT exhibited more intense tissue damage, characterised by tumour cell loss, cells with picnotic nuclei, and extensive fibrosis (Figure 5B).

**Figure 6:**
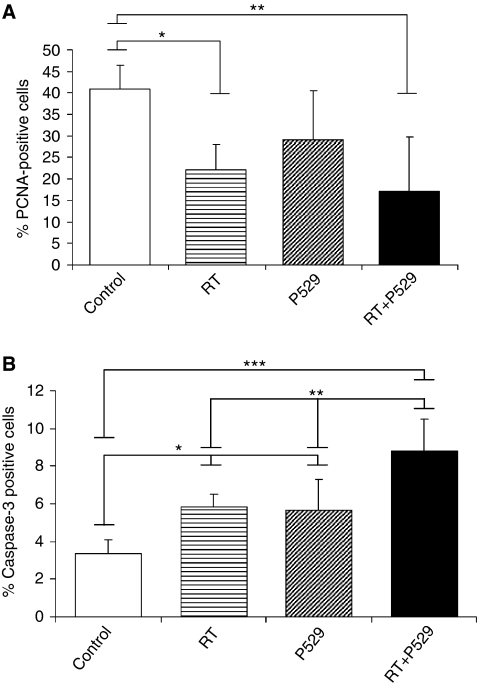
Proliferation and apoptosis in mice subjected to the different treatments. (**A**) The percentage of PCNA-positive cells is significantly reduced in RT- and RT+P529-treated mice, compared with control mice. (**B**) The proportion of active caspase-3-positive cells in P529, RT, and especially in RT+P529, is significantly increased with respect to controls. ^*^P<0.05; ^**^P<0.01; ^***^P<0.001.

Proliferation and apoptotic rates were also calculated in these tumours. In controls, 40.9±5.5% of tumour cells were PCNA-positive (Figure 6A). Proliferation in RT-treated tumours was significantly reduced (*P*<0.05) in comparison with controls. Percentage of PCNA-positive cells in P529-treated mice was 29.1±6.1%, which did not reach statistical difference compared with controls. Interestingly, RT+P529 treatment significantly reduced (*P*<0.01) PCNA-positive cells to 17.1±12.2%. Apoptosis was further assessed quantifying caspase-3-positive cells by immunohistochemistry. Values for controls, RT, P529, and RT+P529 were as follows: 3.3±0.7; 5.8±0.7; 5.6±1.6; and 8.7±1.7%, respectively. Therefore, these data showed that exposure to radiation together with administration of P529 resulted in a significant enhancement of caspase-3-positive cells, compared with individual therapies alone (Figure 6B).

**Figure 7:**
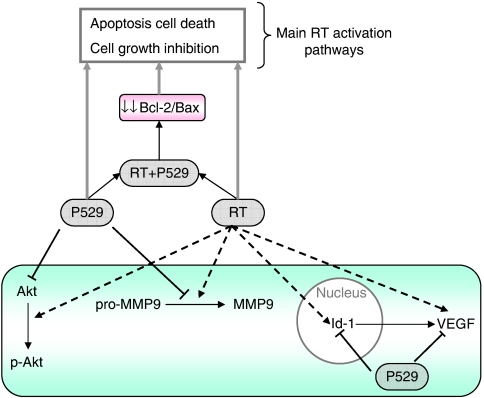
Scheme of the signalling pathways blocked by P529 in PC-3 cells treated with ionising radiation.

Figure 7 depicts a summary of the proposed molecular mechanisms targeted by P529 in PC-3 cells subjected to ionising radiation, according to our results. Our data show that P529 acts through several cell pathways to enhance the therapeutic effect of radiation.

## Discussion

Mortality rates of advanced tumours have not decreased with current therapeutic drugs (Jemal *et al*, 2008). Therefore, new chemical compounds with a higher antitumour activity, alone or in combination with current therapies, are urgently needed. Antioestrogen compounds, such as tamoxifene or clomifene, have shown antitumour and antiangiogenic activities. In this study, we describe the biological activity of 8-(1-Hydroxy-ethyl)-2-methoxy-3-(4-methoxy-benzyloxy)-benzo[*c*]chromen-6-one (P529), a recently discovered drug that targets Akt, which has been shown to inhibit tumour angiogenesis, vascular permeability, and tumour growth, in a mouse model of glioblastoma (Xue *et al*, 2008). P529 is a derivative of 3-hydroxy dibenzo[*b*,*d*]pyran-6-one, which was designed to structurally mimic the antiangiogenic drug 2-methoxyestradiol (2ME_2_) (Schmidt *et al*, 2003). 3-hydroxy dibenzo[*b*,*d*]pyran-6-one exhibits selective antiproliferative activity for endothelial cells (Schmidt *et al*, 2003). We have shown in this study that P529 has a wide spectrum of antitumour efficacy in different tumour cell lines, and significantly enhances the effect of RT in prostate cancer both *in vitro* and *in vivo*.

The structural similarities between dibenzo[*b*,*d*]pyran-6-ones and 2ME_2_, a tubulin polymerisation inhibitor that binds to the colchicine's site, prompted us study such putative activity of P529. Here, we show that P529 neither binds to the colchicine's site nor blocks tubulin polymerisation. Hence, its mode of action is outside of a tubulin agent, thus differing from one of the main 2ME_2_-related mechanisms.

Activation of PI3K/Akt is a critical regulatory signalling pathway involved in cell proliferation, transformation, apoptosis, and angiogenesis (Fang *et al*, 2007). Hyperactivation of PI3K/Akt confers cancer cells resistance to radiation-induced cell death (McKenna and Muschel, 2003; Cheng *et al*, 2006). As P529 is a novel Akt inhibitor (Xue *et al*, 2008) whose antitumour efficacy has been proven *in vivo*, we hypothesised that this drug could enhance the efficacy of RT in prostate cancer. The PC-3 cell line was used because of its aggressiveness and relative resistance to RT (Inayat *et al*, 2002). We have found that P529 reduces dramatically the levels of phospho-Akt induced by radiation exposure (and, in a lesser extent, total Akt), which confirms that Akt is a main target of P529 in cancer cells. The Bcl-2/Bax ratio was also reduced in RT+P529-treated cells, but the effect is likely due to RT alone, as similar levels were found for RT and RT+P529.

Ionising radiation causes DNA breaks and induces a cellular response that activates the tumour suppressor protein p53 (Lain and Lane, 2003). However, in a high percentage of tumours and cancer cells (such as PC-3), the p53 gene is functionally inactive. In addition, upon irradiation, expression of proteins related to cell survival pathways, such as EGF or VEGF/Id-1, are strongly induced (Tsai *et al*, 2007) in a likely attempt to overcome cell stress and reactive oxygen species (ROS) effect. Here, we present the data supporting that the use of P529 could also block the radiation-induced overexpression of survival factors, such as VEGF, Id-1, MMP-2, and MMP-9. The overexpression of Id-1 in tumour cells results in an increased cell proliferation and tumour angiogenesis through the upregulation of VEGF (Ling *et al*, 2006). Id-1 also confers tumour cells resistance to radio- and chemotherapy-induced apoptosis through the activation of PI3K/Akt/NF-*κ*B signalling pathways (Ling *et al*, 2006; Li *et al*, 2007). Interestingly, knockdown of Id-1 strongly sensitises PC-3 cells to chemotherapy-induced cell death (Zhang *et al*, 2006).

Vascular endothelial growth factor is also upregulated in response to ionising radiation (Gorski *et al*, 1999; Gupta *et al*, 2002). Vascular endothelial growth factor not only induces angiogenesis, but also stimulates tumour cell proliferation in an autocrine manner in prostate cancer (Jackson *et al*, 2002). Therefore, targeting VEGF is thought to represent a novel effective way of treating this type of tumour (Rhee and Hoff, 2005). In our study, P529-mediated reduction of VEGF levels is in keeping with the antiproliferative effect of P529 on endothelial cells (HUVECs). Radiation induces MMP-2 (Wang *et al*, 2000) and MMP-9 levels through PI3K/Akt/NF-*κ*B signal transduction pathways as well (Cheng *et al*, 2006), leading to cell invasiveness. Therefore, blockade of MMPs has been proposed as an attractive way to potentiate RT. We have shown that P529 is able to block the activation of MMP-9 and to reduce the levels of MMP-2 and pro-MMP-9 induced by radiation.

Importantly, our *in vivo* experiments have shown that P529 exerts antitumour activity and that the therapeutic efficacy of RT is enhanced by this drug. The anticancer effect is because of a decrease in cell proliferation with a concomitant increase in the number of cells undergoing apoptosis. It is likely that the reduced levels of survival signalling pathways activated by RT in P529-treated mice (i.e., Akt, VEGF, Id-1, and MMPs) end up in a reduced proliferation/apoptosis ratio *in vivo*, leading to tumour shrinkage. We did not observe any toxic effect in mice treated with the drug alone or in combination with RT, as previously shown in a mouse model of glioblastoma (Xue *et al*, 2008).

In conclusion, our results show that the novel compound 8-(1-Hydroxy-ethyl)-2-methoxy-3-(4-methoxy-benzyloxy)-benzo[*c*]chromen-6-one (P529) has a potent antitumour activity in a large variety of tumour cells. Moreover, P529 enhances the effect of RT in PC-3 prostate cancer cells. Pathways involving Akt, VEGF, Id-1, MMP-9, MMP-2, and Bcl-2/Bax are targeted by this novel drug. The ability to act at different pathway levels (mainly the Akt pathway), all of them involved in the response to radiation, makes this compound an attractive agent that might limit the possible tumour escaping routs. Our results suggest that this novel compound could be tested in the future in the clinic as a novel anticancer therapy to enhance the effect of RT.

## Figures and Tables

**Table 1:** Cell growth inhibition 50 (GI50) of P529 in the NCI-60 tumor cell lines panel

**Panel/cell line**	**GI50 (*μ*M)**
*Leukemia*	
CCRF-CEM	
HL-60(TB)	2.28
K-562	4.11
MOLT-4	32.7
RPMI-8226	21.5
SR	2.14
	
*Non-small cell lung cancer*	
A549/ATCC	13.1
EKVX	22.3
HOP-62	11.2
NCI-H226	21.2
NCI-H23	15.1
NCI-H322M	34.3
NCI-H460	11.4
NCI-H522	8.84
	
*Colon cancer*	
COLO 205	23.7
HCC-2998	12.1
HCT-116	10.8
HCT-15	12.9
HT29	6.04
KM12	14.2
SW-620	14.5
	
*CNS cancer*	
SF-268	6.09
SF-295	12.2
SF-539	14.7
SNB-19	11.2
SNB-75	5.18
U251	12
	
*Melanoma*	
LOX IMVI	22.5
MALME-3M	17.2
M14	16.4
SK-MEL-2	18.6
SK-MEL-26	14.5
SK-MEL-5	9.12
UACC-257	15.4
UACC-62	12.1
	
*Ovarian cancer*	
ICROV1	14.9
OVCAR-3	12.8
OVCAR-4	12.8
OVCAR-5	24.1
OVCAR-8	9.02
SK-OV-3	13.6
	
*Renal cancer*	
786-0	16.2
A496	15.9
ACHN	13.3
CAKI-1	11.2
RXF 393	5.24
SN 12C	17
TK-10	18.2
UO-31	15.5
	
*Prostate cancer*	
PC-3	12.4
DU-145	18.6
	
*Breast cancer*	
MCF7	6.01
NCI/ADR-RES	12.6
MDA-MB-231/ATCC	14.7
HS 578T	13.3
MDA-MB-435	2.64
BT-549	17

**Table 2:** Tubulin binding to the colchicine site assay

**Sample**	**CPM**	**% Inhibition**
Blank (no tubulin)	1159	—
Tubulin (no inhibitor)	18384	—
1 *μ*M CA4P	4288	82
5 *μ*M CA4P	1961	97
5 *μ*M 2ME2	7624	62
50 *μ*M 2ME2	3256	88
5 *μ*M Palomid 529	17 036	8
50 *μ*M Palomid 529	17 129	7

The assay shows displacement of 5 *μ*M [^3^H]colchicine on 1 *μ*M tubulin by test agents. Combretastatin-A4-phosphate (CA4P): a microtubule-destabilizing agent. CPM: counts per minute.
